# Combining high throughput and high quality for cryo-electron microscopy data collection

**DOI:** 10.1107/S2059798320008347

**Published:** 2020-07-27

**Authors:** Felix Weis, Wim J. H. Hagen

**Affiliations:** a The Cryo-Electron Microscopy Service Platform, Structural and Computational Biology Unit, European Molecular Biology Laboratory, Heidelberg, Germany

**Keywords:** cryo-electron microscopy, coma-free imaging, fringe-free imaging, high-end data collection

## Abstract

Cryo-electron microscopy (cryo-EM) can be used to elucidate the 3D structure of macromolecular complexes. Driven by technological breakthroughs in electron-microscope and electron-detector development, coupled with improved image-processing procedures, it is now possible to reach high resolution both in single-particle analysis and in cryo-electron tomography and subtomogram-averaging approaches. As a consequence, the way in which cryo-EM data are collected has changed and new challenges have arisen in terms of microscope alignment, aberration correction and imaging parameters. This review describes how high-end data collection is performed at the EMBL Heidelberg cryo-EM platform, presenting recent microscope implementations that allow an increase in throughput while maintaining aberration-free imaging and the optimization of acquisition parameters to collect high-resolution data.

## Abstract 2

Methods for obtaining the maximum out of a cryo-EM data-collection session are described.

## 1. Introduction   

Cryogenic transmission electron microscopy (cryo-EM) can be used to elucidate the 3D structure of macromolecular complexes. The sample is embedded in a thin layer of vitreous ice and maintained at liquid-nitrogen temperature. It is then imaged directly in the microscope and a 3D reconstruction may be calculated either from the projections of individual macromolecular complexes by determining their orientations in the case of single-particle analysis (SPA; Lyumkis, 2019 ▸) or by averaging the 3D reconstructions of the complex within tomograms in the case of cryo-electron tomography (cryo-ET) and subtomogram averaging (STA) (Schur, 2019 ▸). For many years cryo-EM was limited to low resolution, but recent advances have made it one of the main methods of choice for the determination of high-resolution structures, which has been termed ‘The Resolution Revolution’ (Kühlbrandt, 2014 ▸). Sub-3 Å resolution structures are now released almost every week and sub-2 Å resolution structures have also been reported for SPA (Tan *et al.*, 2018 ▸; Zivanov *et al.*, 2018 ▸; Weis *et al.*, 2019 ▸), while sub-5 Å resolution structures show the great potential of cryo-ET and STA (Schur *et al.*, 2016 ▸; Mattei *et al.*, 2018 ▸; Hutchings *et al.*, 2018 ▸). There are several contributors to this progress. The first is the development of a new generation of electron detectors that record images with unprecedented quality. The second is the availability of highly stable and fully automated electron microscopes allowing long unattended operation and automated data collection. The last is the simultaneous development of improved image-processing procedures. The synergy between these breakthroughs has led to cryo-EM structures with unrivalled final resolutions.

The recent developments mentioned in the previous paragraph have significantly modified the way that cryo-EM data are collected. Currently, after only a few hours of setup, data collection can run in a fully unattended and automated way for several days, producing a high-quality data set of thousands of micrographs or hundreds of tomograms. These improvements have led to new challenges in terms of session setup, microscope alignments and acquisition parameters, and new strategies were required to tackle these questions. This communication will present the key features of the high-end data-collection workflow performed on the Titan Krios G3i (Thermo Fisher Scientific; TFS) equipped with a Quantum K2 camera (Gatan) at the EMBL Heidelberg cryo-EM platform: (i) fine-tuning of the objective lens for aberration-free imaging, (ii) microscope alignments allowing a small electron beam and aberration-free beam shift/image shift, (iii) the pixel size for optimized data collection and (iv) the relation between defocus and the expected resolution.

## 2. Recent microscope optics implementations   

### 2.1. Objective lens tuning is required for aberration-free imaging   

SPA and STA approaches now allow researchers to reach sub-4 Å resolution. At such a resolution, small amounts of beam tilt can result in a significant amount of axial coma, a resolution-limiting aberration, and current–axis alignments are not accurate enough to ensure high-quality phase information in the recorded images (Glaeser *et al.*, 2011 ▸). It is therefore necessary to finely tune the beam tilt by running an (axial) coma-free alignment procedure using the Zemlin tableau (Zemlin *et al.*, 1978 ▸). All of the current–axis alignments (called ‘direct alignments’ in the TFS microscope user interface) can be considered as stable enough not to be performed for every acquisition session, but the coma-free alignment procedure is performed each time before running a data collection. Indeed, the amount of beam tilt, and thus the amount of coma, is different according to the magnification and the size of the beam used for data recording, meaning that it has to be redone if one of these parameters changes.

In practice, the same alignment file is loaded at the beginning of each session and the imaging parameters (magnification, dose rate/spot size and beam size) are chosen by making sure the beam size is in the parallel illumination range as stated in the ‘beam settings’ panel of the user interface. The coma-free alignment procedure is then performed using automated software subroutines, for instance from TFS or *SerialEM* (Mastronarde, 2005 ▸), followed by the insertion and manual centering of an objective aperture and an objective astigmatism correction (again using an automated software subroutine). Any subsequent adjustment of the electron dose should preferably be performed by modifying the exposure time rather than the spot size. Changing the spot size introduces a very limited amount of beam tilt, while changing the beam size introduces a larger amount of beam tilt, making the coma-free procedure invalid.

### 2.2. Fringe-free illumination and active beam-tilt compensation allow higher throughput while maintaining coma-free imaging   

**Figure 1:**
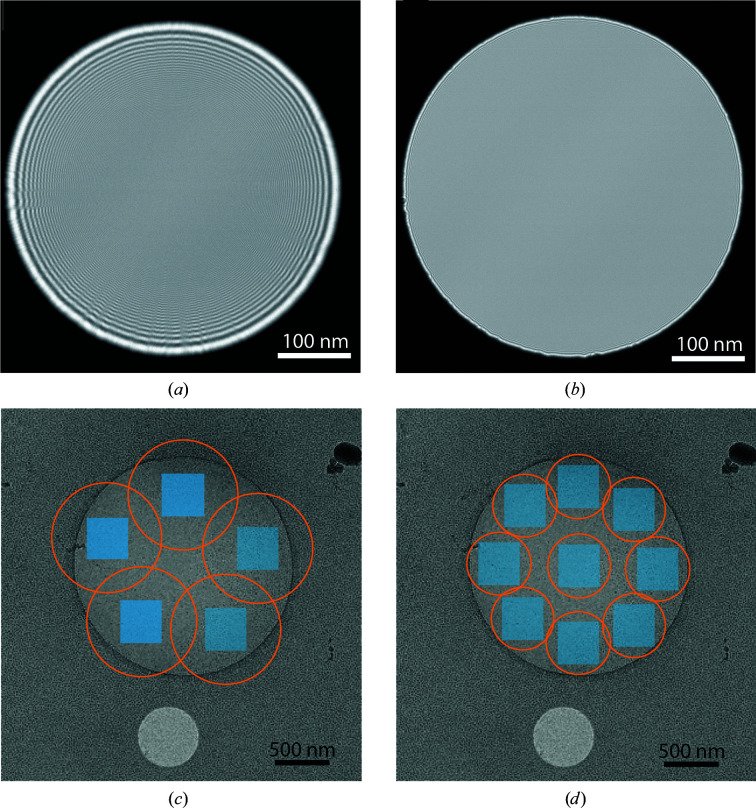
Fringe-free illumination. (*a*, *b*) Image of the beam without (*a*) and with (*b*) fringe-free illumination. The beam diameter is 460 nm and the images were recorded at spot 9 over a 20 s exposure with a pixel size of 1.34 Å. (*c*, *d*) Acquisition scheme without (*c*) and with (*d*) fringe-free illumination. The sample is embedded in a thin layer of ice over a holey carbon film with 2 µm diameter holes. The blue squares represent the imaged area in the context of a 1.04 Å pixel. Without fringe-free illumination (*c*) the beam size, depicted by an orange circle, needs to be ∼1 µm in diameter in order to avoid seeing fringes within the imaged area, limiting the number of acquisitions to five within the hole. In the case of fringe-free illumination (*d*), a beam size of 600 nm is enough to cover the camera, allowing up to ten acquisitions.

In a Titan Krios, the C2 condenser aperture is not in the C2 condenser lens plane, triggering a Fresnel (near-field) diffraction pattern of the beam that is seen as fringes (Fig. 1 ▸ *a*). As a consequence, to avoid seeing the fringes in the recorded images, the beam needs to be made roughly twice the size of the camera, exposing large sample areas and limiting the number of acquisition positions (Fig. 1 ▸ *c*). However, this phenomenon can be avoided by using a recent TFS development: fringe-free illumination (Fig. 1 ▸ *b*; Konings *et al.*, 2019 ▸). The principle is to image the C2 aperture, instead of the C2 lens plane, on the sample plane. Practically speaking, it involves tuning the objective lens coupled with a mechanical adjustment of the stage height and is usable over the whole range of magnifications used for SPA and cryo-ET. It is therefore possible to use a very tight beam around the detector without seeing any fringes on the recorded images and to fit more records in the same area of interest (Fig. 1 ▸ *d*).

In SPA, it is not recommended to move the stage for each acquisition point, otherwise one needs to wait each time for the stage to stabilize, dramatically reducing the data-collection throughput. To collect data as rapidly as possible, the current strategy is to deflect the beam to the successive acquisition positions (beam shift/image shift). The drawback is that the beam is tilted and moves away from the coma-free axis (see Section 2.1), inducing a certain amount of off-axis coma directly related to the direction of the deflection and the distance from the coma-free axis (Christenson & Eades, 1986 ▸). By adjusting the tilt angle at each new position, this off-axis coma can be avoided (Eades, 2006 ▸). The dynamic compensation for the position-dependence of the coma can be used to collect data from positions that are several micrometres away from the coma-free axis and can also be used for the hole-centring step before actual acquisitions within this hole, limiting the number of stage movements to only one per position. There are now several automated implementations of this procedure: that from TFS, *Leginon* (Suloway *et al.*, 2005 ▸), that of Wu *et al.* (2019 ▸) and *SerialEM* (Mastronarde, 2005 ▸). The latter is usable on all microscopes without using parallel illumination (with a new calibration per illumination setting). Another advantage is that the images can be considered as off-axis coma-free and allow solution using only one beam tilt for the whole data set in *RELION* (Zivanov *et al.*, 2018 ▸) or *cryoSPARC* (Punjani *et al.*, 2017 ▸).

## 3. Optimization of acquisition parameters   

### 3.1. Smaller pixel size leads to better data   

An important parameter that needs to be chosen for the recorded images is the magnification, as it directly determines the pixel size, the field of view and the size of the beam, given that the latter is kept as small as possible to cover the camera. It is important to note that in the case of SPA acquisition, the magnification hardly affects the throughput in terms of the number of particles collected in a given amount of time. Indeed, because of the fixed size of the camera chip, using a higher magnification decreases the field of view of the recorded images, but this in turn allows more images to be collected within the same acquisition area (typically a hole), leading to an almost constant imaged area independent of the magnification (see Table 1 ▸). Furthermore, since the recommended electron dose rate at the camera level is fixed (2–10 electrons per pixel per second for the K2 camera operated in counting mode), using a higher magnification decreases the time length of each individual exposure given that the total electron dose is kept the same at the sample level, giving a similar overall acquisition time for different magnifications (see Table 1 ▸).

A study by Stagg *et al.* (2008 ▸) suggests that collecting data at higher magnification is highly beneficial for the overall quality of the resulting 3D reconstruction. It reports that the resolution increases with higher magnification for a given number of particles by offsetting the high-frequency dampening effects of the detector. Since data collection at higher magnification does not decrease the throughput in terms of number of particles, it is then recommended to collect data with a small pixel size even though the expected final resolution is far from the theoretical maximum resolution (the Nyquist frequency; two times the pixel size). A drawback of using a small pixel size is that the box size (in pixels) used during the processing will be larger, increasing the computational resources needed, but nowadays most processing workflows use highly binned data for the initial rounds of 2D and 3D classification and 3D refinement, meaning that only the actual fraction of the particles used for the final 3D refinement step is actually needed at full size.

### 3.2. The closer to focus the better   

Applying a certain amount of defocus to the images during acquisition to add phase contrast is the prevalent method in most of the current acquisition routines. An effect of defocus (Δ*F*) is to displace image spacings of resolution *d* by a distance *R* = λΔ*F*/*d*, where λ is the electron wavelength (Rosenthal & Henderson, 2003 ▸; de Jong & Van Dyck, 1993 ▸). Since most of the processing programs correct for the contrast transfer function (CTF) on the extracted particles, it is important to make sure that all of the displaced information is still included in the boxed image. Given that the recommended box size for extraction and processing is approximately two times larger than the longest axis of the particle (*D*), we can calculate the highest defocus that one should apply by using the formula described by Rosenthal & Henderson (2003 ▸), box size = *D* + 2*R*, considering that the maximum resolution spacing expected, *d*, is two times the pixel size (see Table 2 ▸ for values calculated from various test samples). For all samples, these values are very low (below 1 µm defocus for most of them) and quite far from the usual defocus range found in the literature: up to 4 µm or more. The work of Tan and coworkers on the AAV2 capsid shows a decrease in the resolution of the reconstruction when using a defocus of greater than 1.4 µm with a small box size (Tan *et al.*, 2018 ▸). This statement of course needs to be balanced by the fact that the vast majority of the samples will not reach the theoretical maximum resolution at such small pixel sizes and also that small samples can need a significant amount of phase contrast, and hence defocus, to be visible, especially if the ice thickness and/or the buffer composition (Drulyte *et al.*, 2018 ▸) are not optimal.

In the cases where high resolution (sub-3 Å) is needed (ligand-binding studies, drug discovery *etc.*) the strategy is then to collect as close to focus as possible and to process the data with a box size adapted to the resolution expected, at least in the final 3D refinement steps. One drawback of using a large box size is the associated computational cost, which can drastically slow down the processing or even make it impossible for most of the current popular GPU setups, although some recent CPU optimization developments have been made in *RELION* to deal with large box sizes (Zivanov *et al.*, 2018 ▸).

## 4. Conclusion   

The automation of coma-free alignment coupled with the use of one direct alignments file drastically facilitates the setup of data collection on a high-end microscope, allowing more independent users to run the microscopes and enhancing the overall efficiency of the cryo-EM platform, given the ever-increasing popularity of the method. The implementation of fringe-free imaging and aberration-free image shift increases the throughput, reducing the length of the session and consequently the cost, given the high cost of data collection on a high-end microscope. It is also of primary importance to carefully select the acquisition parameters (pixel size and defocus range) according to the expected/desired resolution in order to get the maximum out of the data-collection session. We now routinely collect SPA data sets using active beam-tilt compensation and fringe-free imaging at a pixel size of ∼0.8 Å (Wandzik *et al.*, 2020 ▸; Qi, Di Minin *et al.*, 2019 ▸; Qi, Sorrentino *et al.*, 2019 ▸; Cannac *et al.*, 2020 ▸; Flaugnatti *et al.*, 2020 ▸; Zivanov *et al.*, 2018 ▸; Muir *et al.*, 2020 ▸; Oosterheert *et al.*, 2018 ▸) or even smaller (Weis *et al.*, 2019 ▸). The ability to collect more/better data in a shorter time allows increasingly dynamic, flexible and heterogeneous complexes to be imaged (time-resolved studies, mechanistic studies *etc.*), as they require a large number of particles for classification in order to clearly distinguish subpopulations of interest.

## Figures and Tables

**Table 1:** Comparison of acquisition parameters for different magnifications

Magnification	×105 000	×130 000	×165 000
Pixel size (Å)	1.34	1.04	0.81
Beam size (diameter) (nm)	750	600	450
No. of shots per 2 µm diameter hole	5	9	15
Area imaged (considering a K2 camera, 3838 × 3710 pixels) (µm^2^)	∼1.3	∼1.4	∼1.4
Electron dose rate at the camera (electrons per pixel per second)	4	4	4
Total electron dose at the sample (e^−^ Å^−2^)	∼40	∼40	∼40
Exposure time per position (s)	∼18	∼10	∼6
Total exposure time per hole (s)	∼90	∼90	∼90

**Table 2:** Box size and optimal defocus for different pixel sizes

Sample	Methemoglobin (∼64 kDa)	Apoferritin (∼480 kDa)	β-Galactosidase (∼460 kDa)	70S ribosome (∼2.7 MDa)
Size (nm)	6.5	12	18	25
Pixel size (Å)	0.81/1.04/1.34	0.81/1.04/1.34	0.81/1.04/1.34	0.81/1.04/1.34
Box size (pixels)	162/126/98	298/232/180	446/348/270	618/482/374
Maximum defocus† (µm)	0.27/0.34/0.44	0.49/0.63/0.82	0.74/0.95/1.23	1.03/1.32/1.70

† Calculated using the formula *R* = λΔ*F*/*d* (see Section 3.2). At 300 kV, λ = 1.96876239934912 × 10^−12^ m.
